# Supplementary figures and images for: Autophagy and oxidative stress modulation mediate Bortezomib resistance in prostate cancer
Source: PLoS One. 2024 Feb 27;19(2):e0289904. doi: 10.1371/journal.pone.0289904 (PMC10898778; doi:10.1371/journal.pone.0289904)

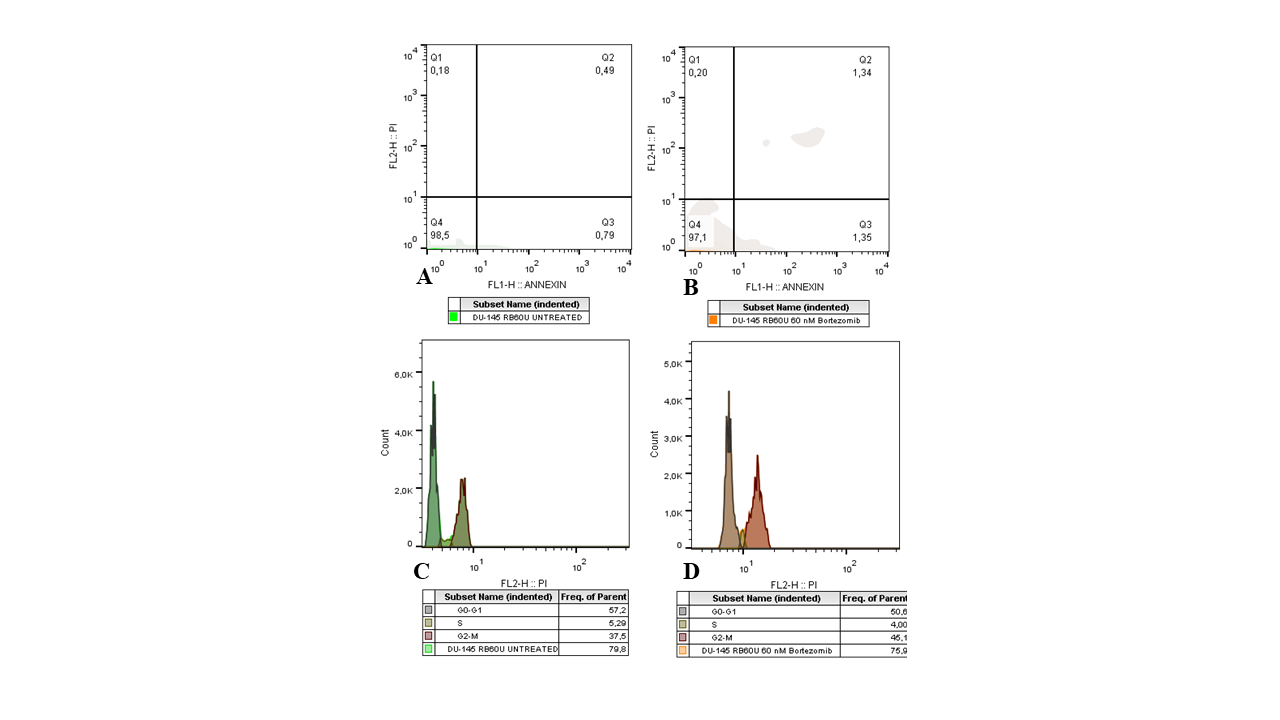

Supplement: S1 Fig — (A, B) The DU-145 RB60U cell clone maintains the apoptosis evasion observed on the DU-145 RB60 cells after a 24-week drug deprivation with a slight increase of its apoptotic rate. (C, D) Following the same procedures as in naïve and DU-145 RB60 cells, the cell cycle of DU-145 RB60U cells was analyzed using PI and indicated a mild G2 arrest following incubation with 60 nM of Bortezomib for the first time after 24 weeks. The subsequent cell cycle inhibition does not result in apoptosis as has been shown by the rest experiments assessing apoptosis compared to the naïve DU-145 that within 48–72 h undergo apoptosis. (TIF) [file pone.0289904.s001.tif]

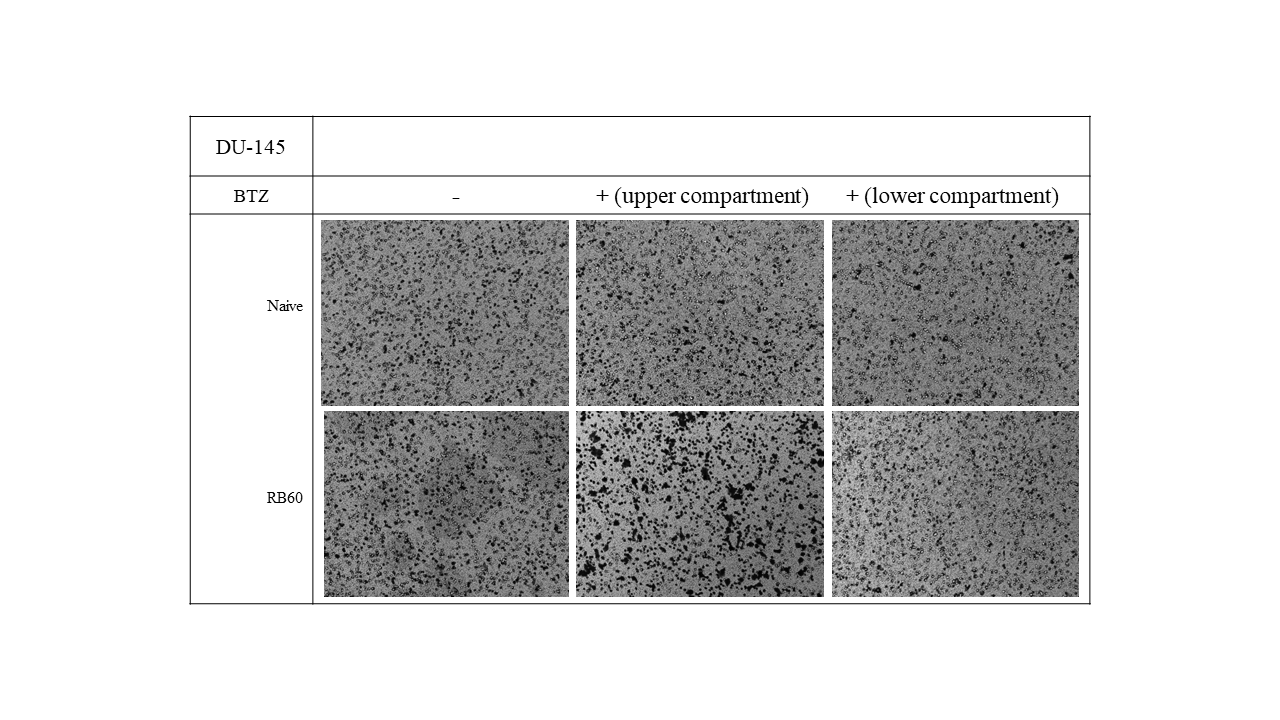

Supplement: S2 Fig — Cells were transferred into a chamber containing serum-free medium with or without Bortezomib. The chambers were placed inside microplates’ wells containing medium supplemented with 20% FBS and left to migrate for 24 h. The DU-145 cells, when exposed to Bortezomib (20 nM), decreased their migration rate. Inhibition of migration was also observed when Bortezomib was added to the lower compartment, indicating a chemorepellent role. The DU-145 RB60 cells were also repelled by Bortezomib (60 nM), while the presence of the drug in the upper compartment induced migration towards the other side of the membrane, where Bortezomib was absent. (TIF) [file pone.0289904.s002.tif]

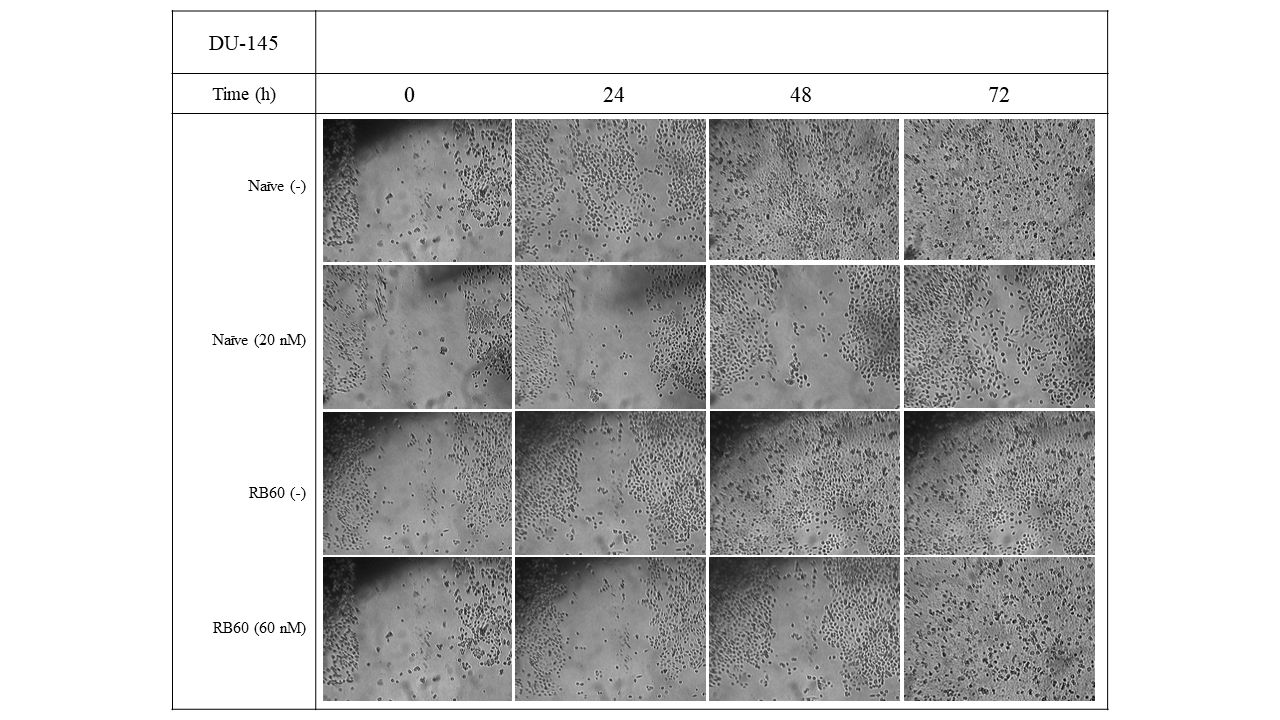

Supplement: S3 Fig — Cells were seeded on 6-well plates and left to form monolayers. After reaching the desired confluency, wounds were scratched, and the Bortezomib-free media were replaced with medium containing 10% FBS and Bortezomib. The naïve cells were assessed using 20 nM of Bortezomib, and the DU-145 RB60 cells were assessed under the influence of 60 nM Bortezomib. Compared to the control group, the DU-145 naïve cells’ ability to heal wounds was heavily impaired by Bortezomib, while the same effect was not observed on the DU-145 RB60 cells. The resistant cells were able to completely heal the scratches after 72 h of incubation, and the same was achieved by the untreated naïve cells. (TIF) [file pone.0289904.s003.tif]
