# Supplementary material for: Autophagy and oxidative stress modulation mediate Bortezomib resistance in prostate cancer
Source: PLoS One. 2024 Feb 27;19(2):e0289904. doi: 10.1371/journal.pone.0289904 (PMC10898778; doi:10.1371/journal.pone.0289904)
Supplement: S1 Raw images — (PDF) [file pone.0289904.s004.pdf]

Western Blot

Autophagy and Oxidative Stress Modulation Mediate Bortezomib Resistance in Prostate Cancer

Kalliopi Zafeiropoulou[1,2]¶, Georgios Kalampounias[1]¶, Spyridon Alexis[2], Daniil Anastasopoulos[1], Argiris Symeonidis[2] and Panagiotis Katsoris[1]*

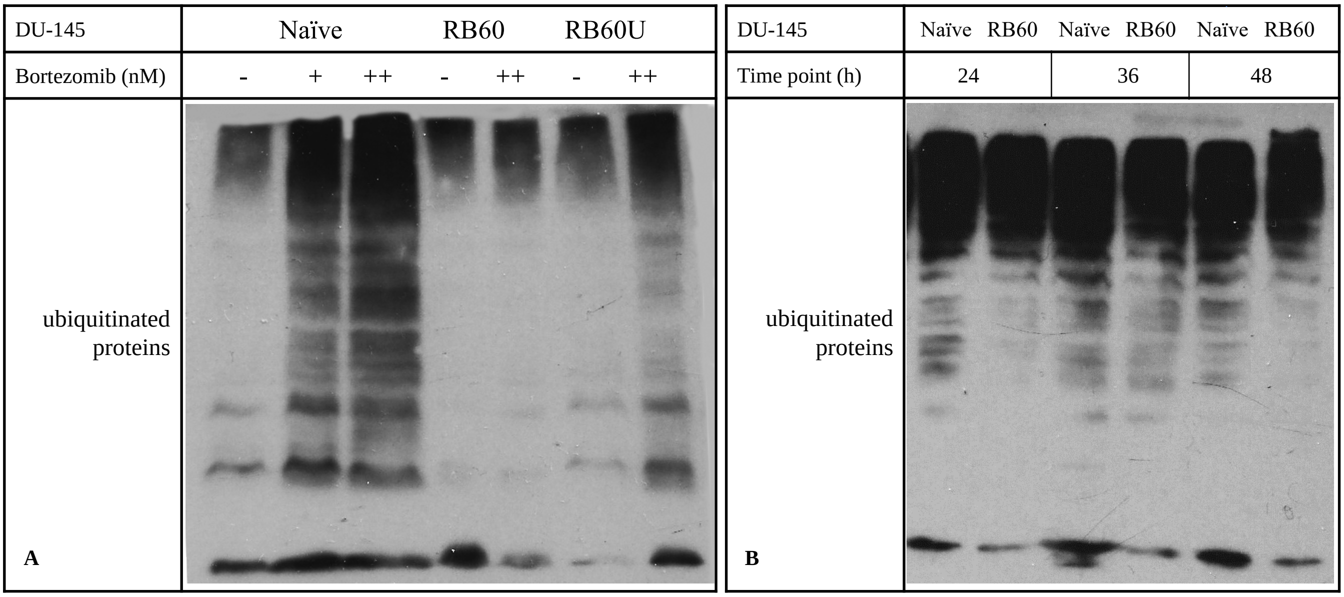

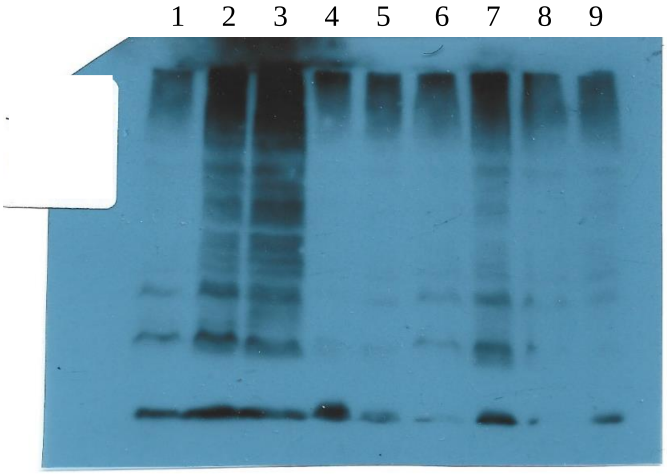

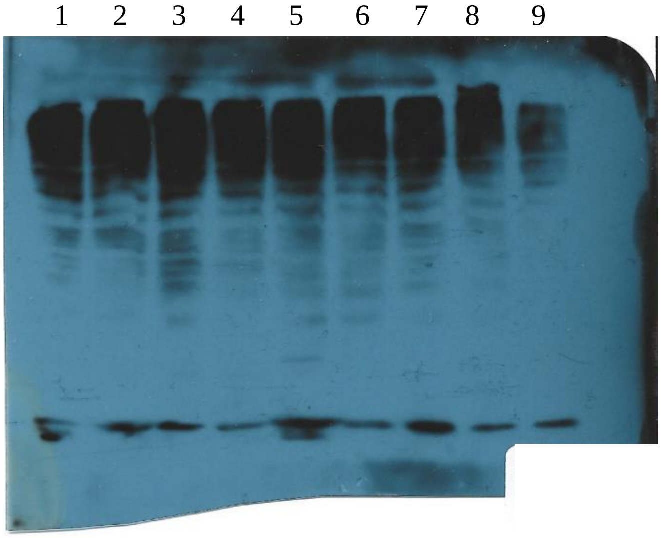

1→ DU-145 naïve Untreated
2→ DU-145 naïve 20 nM BTZ 24h
3→ DU-145 naïve 60 nM BTZ 24 h
4→ DU-145 RB60 Untreated 24 h
5→ DU-145 RB60 60 nM BTZ 24 h
6→ DU-145 RB60U Untreated
7→ DU-145 RB60U 60 nM BTZ 24 h
8→ X
9→ X

1→ X
2→ X
3→ DU-145 Naive 60 nM BTZ 24 h
4→ DU-145 RB60 60 nM BTZ 24 h
5→ DU-145 Naive 60 nM BTZ 36 h
6→ DU-145 RB60 60 nM BTZ 36 h
7→ DU-145 Naïve 60 nM BTZ 48 h
8→ DU-145 RB60 60 nM BTZ 48 h
9→ X

DU-145	RB60	N
Bortezomib (nM)	60 120 180 20	
ubiquitinated proteins	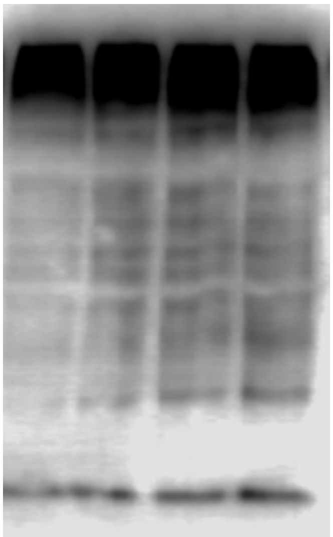	

This image was captured using a LI-COR Western Blot Acquisition System from a partner's laboratory at the University of Patras and was saved as .tif file

DU-145	Naïve		RB60	
Bortezomib	+	-	++	-
PSMB5	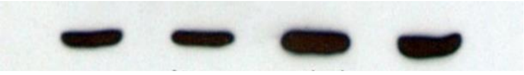			

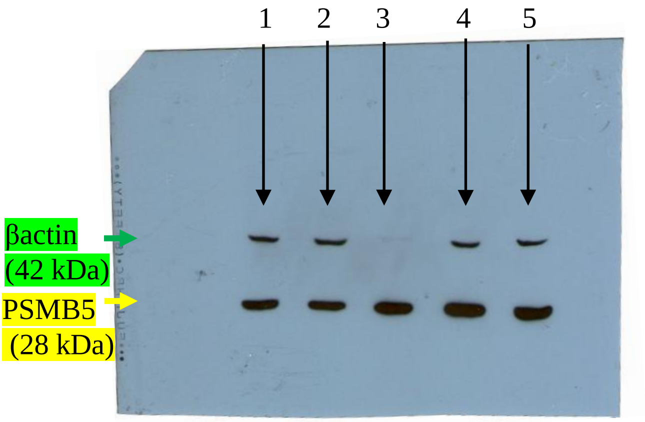

1→ DU-145 naïve 20 nM BTZ 24 h
2→ DU-145 naïve Untreated
3→ X (cropped out)
4→ DU-145 RB60 60 nM
5→ DU-145 RB60 Untreated 24 h

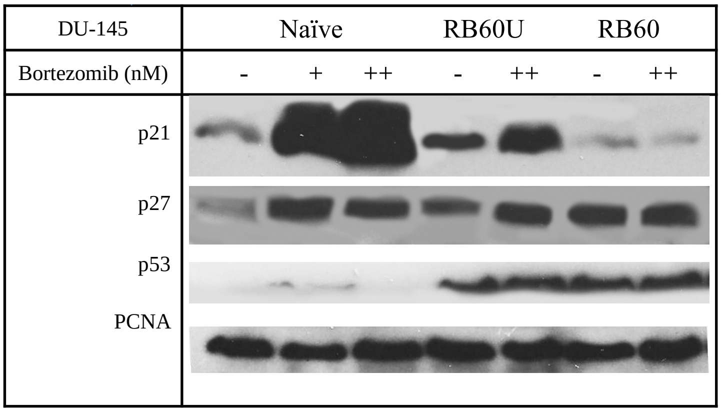

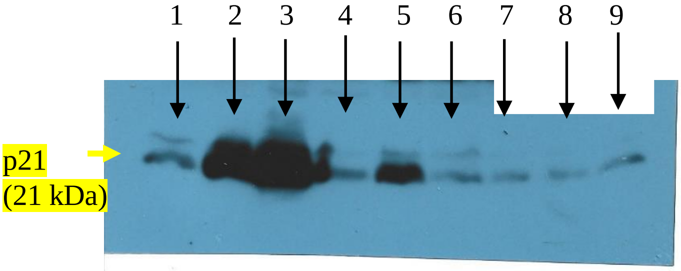

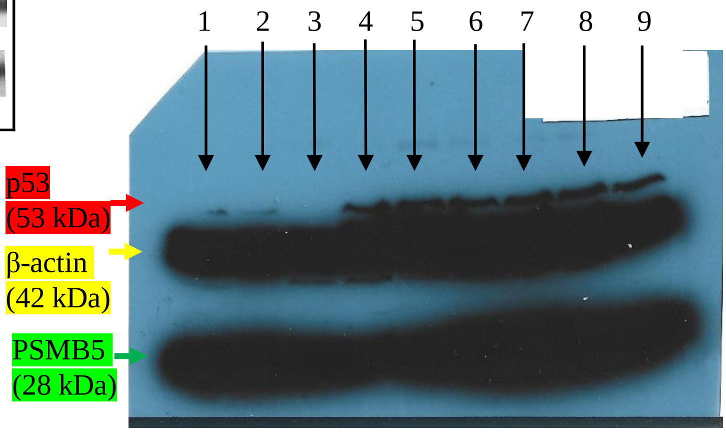

1→ DU-145 naïve Untreated
2→ DU-145 naïve 20 nM BTZ 24h
3→ DU-145 naïve 60 nM BTZ 24 h
4→ DU-145 RB60U Untreated
5→ DU-145 RB60U 60 nM BTZ 24 h
6→ DU-145 RB60 Untreated 24 h
7→ DU-145 RB60 60 nM BTZ 24 h
8→ X
9→ X

1→ DU-145 naïve Untreated
2→ DU-145 naïve 20 nM BTZ 24h
3→ DU-145 naïve 60 nM BTZ 24 h
4→ DU-145 RB60U Untreated
5→ DU-145 RB60U 60 nM BTZ 24 h
6→ DU-145 RB60 Untreated 24 h
7→ DU-145 RB60 60 nM BTZ 24 h
8→ X
9→ X

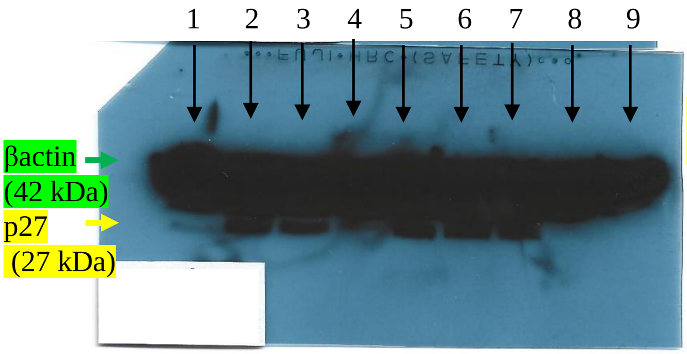

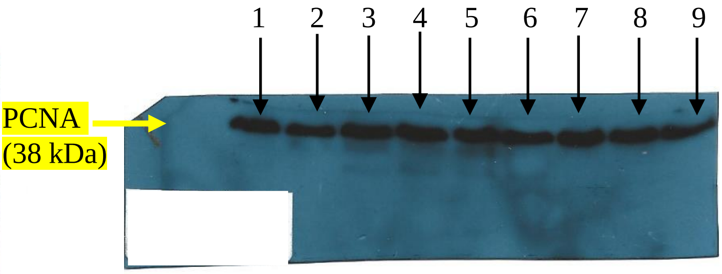

1→ DU-145 naïve Untreated
2→ DU-145 naïve 20 nM BTZ 24h
3→ DU-145 naïve 60 nM BTZ 24 h
4→ DU-145 RB60U Untreated
5→ DU-145 RB60U 60 nM BTZ 24 h
6→ DU-145 RB60 Untreated 24 h
7→ DU-145 RB60 60 nM BTZ 24 h
8→ X
9→ X

1→ DU-145 naïve Untreated
2→ DU-145 naïve 20 nM BTZ 24h
3→ DU-145 naïve 60 nM BTZ 24 h
4→ DU-145 RB60U Untreated
5→ DU-145 RB60U 60 nM BTZ 24 h
6→ DU-145 RB60 Untreated 24 h
7→ DU-145 RB60 60 nM BTZ 24 h
8→ X
9→ X

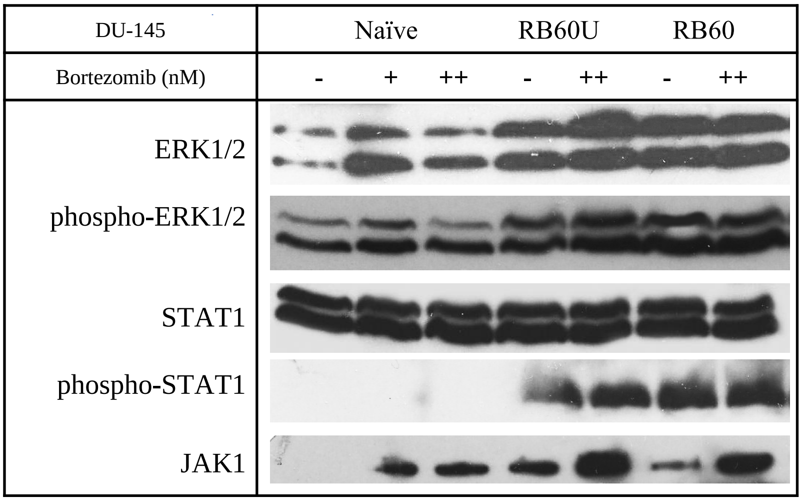

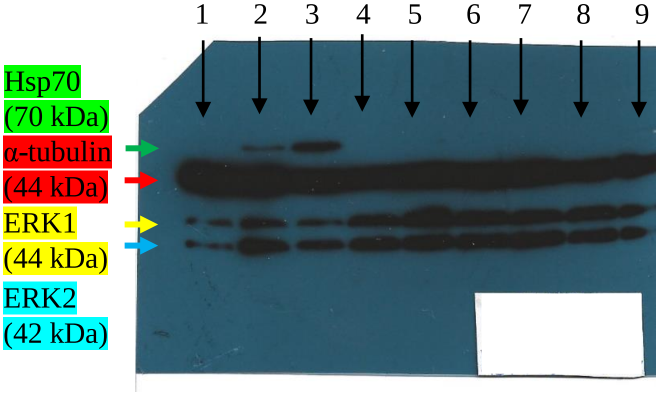

1→ DU-145 naïve Untreated
2→ DU-145 naïve 20 nM BTZ 24h
3→ DU-145 naïve 60 nM BTZ 24 h
4→ DU-145 RB60U Untreated
5→ DU-145 RB60U 60 nM BTZ 24 h
6→ DU-145 RB60 Untreated 24 h
7→ DU-145 RB60 60 nM BTZ 24 h
8→ X
9→ X

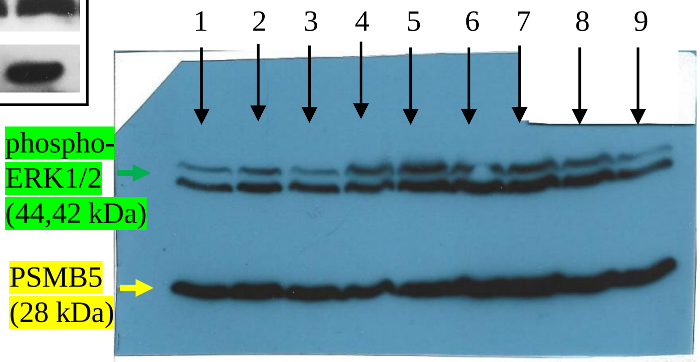

1→ DU-145 naïve Untreated
2→ DU-145 naïve 20 nM BTZ 24h
3→ DU-145 naïve 60 nM BTZ 24 h
4→ DU-145 RB60U Untreated
5→ DU-145 RB60U 60 nM BTZ 24 h
6→ DU-145 RB60 Untreated 24 h
7→ DU-145 RB60 60 nM BTZ 24 h
8→ X
9→ X

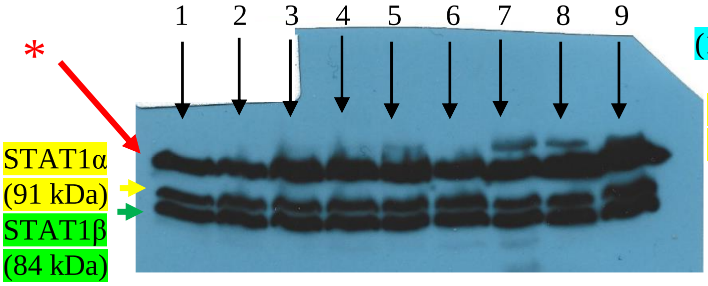

1→ DU-145 naïve Untreated
2→ DU-145 naïve 20 nM BTZ 24h
3→ DU-145 naïve 60 nM BTZ 24 h
4→ DU-145 RB60U Untreated
5→ DU-145 RB60U 60 nM BTZ 24 h
6→ DU-145 RB60 Untreated 24 h
7→ DU-145 RB60 60 nM BTZ 24 h
8→ X
9→ X

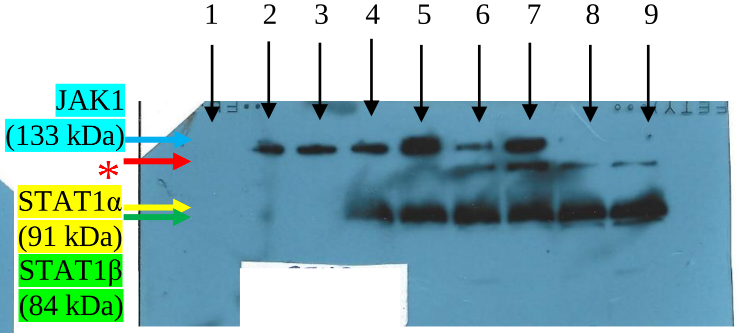

1→ DU-145 naïve Untreated
2→ DU-145 naïve 20 nM BTZ 24h
3→ DU-145 naïve 60 nM BTZ 24 h
4→ DU-145 RB60U Untreated
5→ DU-145 RB60U 60 nM BTZ 24 h
6→ DU-145 RB60 Untreated 24 h
7→ DU-145 RB60 60 nM BTZ 24 h
8→ X
9→ X

* This one is signal from the kinase FAK (125 kDa) which was blotted as a test but is not contained in our paper's results. NaN_3 had been used to block the signal from the secondary antibody because the membrane was reprobed; however, some signal leakgage was observed. Due to hardships detecting FAK and other molecules of its pathway, we did not further examine it. Therefore, is does not appear on our paper

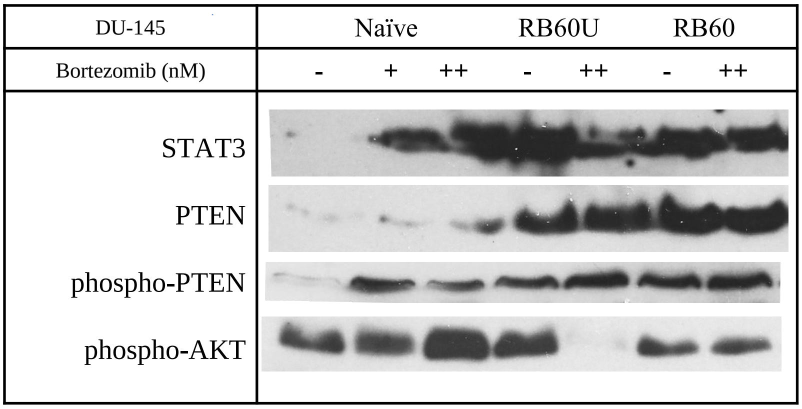

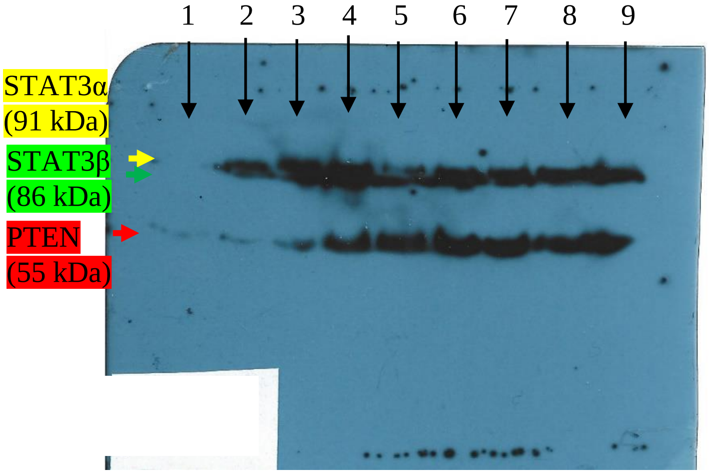

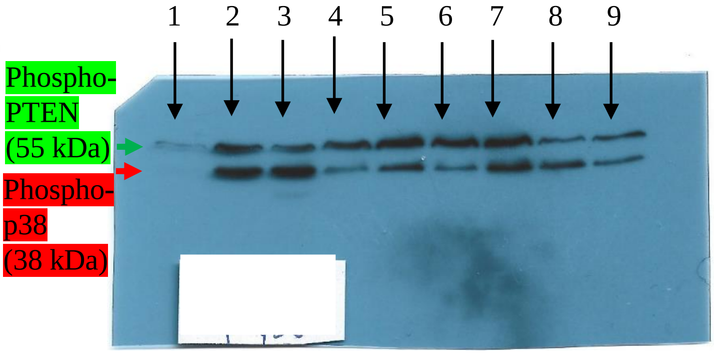

1→ DU-145 naïve Untreated
2→ DU-145 naïve 20 nM BTZ 24h
3→ DU-145 naïve 60 nM BTZ 24 h
4→ DU-145 RB60U Untreated
5→ DU-145 RB60U 60 nM BTZ 24 h
6→ DU-145 RB60 Untreated 24 h
7→ DU-145 RB60 60 nM BTZ 24 h
8→ X
9→ X

1→ DU-145 naïve Untreated
2→ DU-145 naïve 20 nM BTZ 24h
3→ DU-145 naïve 60 nM BTZ 24 h
4→ DU-145 RB60U Untreated
5→ DU-145 RB60U 60 nM BTZ 24 h
6→ DU-145 RB60 Untreated 24 h
7→ DU-145 RB60 60 nM BTZ 24 h
8→ X
9→ X

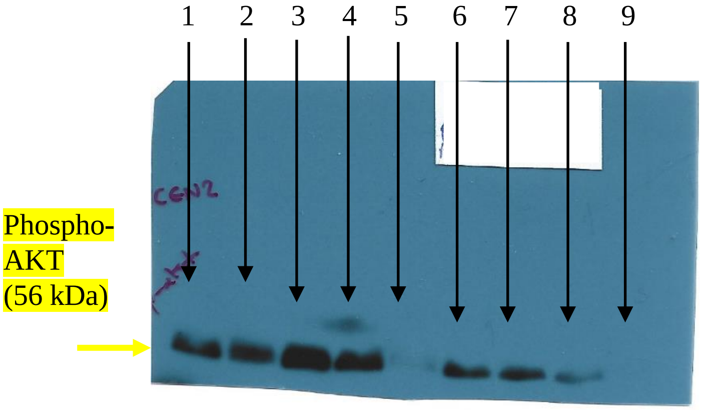

1→ DU-145 naïve Untreated
2→ DU-145 naïve 20 nM BTZ 24h
3→ DU-145 naïve 60 nM BTZ 24 h
4→ DU-145 RB60U Untreated
5→ DU-145 RB60U 60 nM BTZ 24 h
6→ DU-145 RB60 Untreated 24 h
7→ DU-145 RB60 60 nM BTZ 24 h
8→ X
9→ X

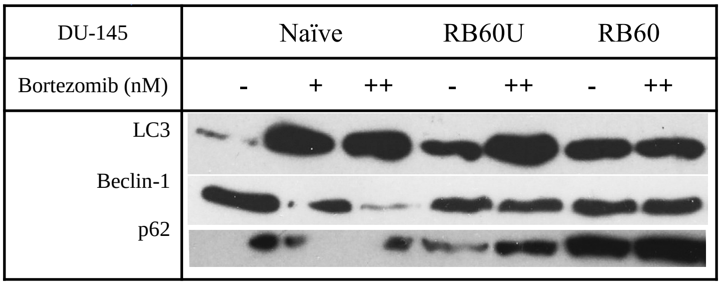

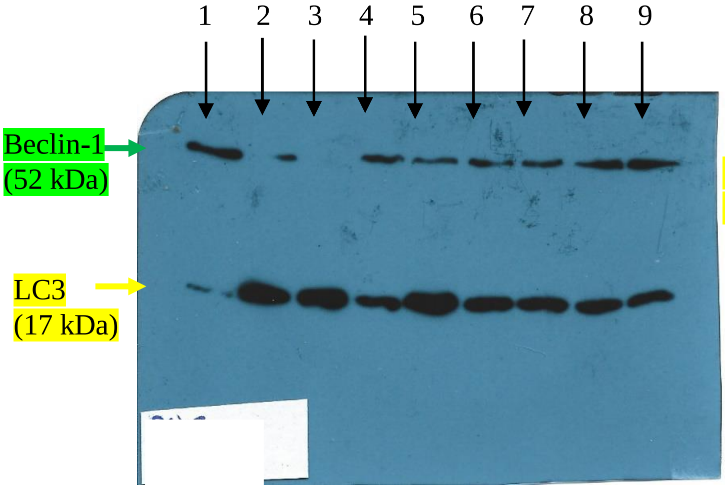

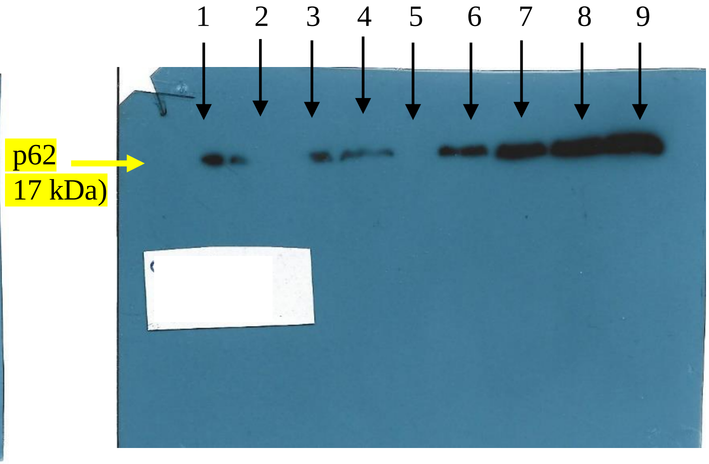

1→ DU-145 naïve Untreated
2→ DU-145 naïve 20 nM BTZ 24h
3→ DU-145 naïve 60 nM BTZ 24 h
4→ DU-145 RB60U Untreated
5→ DU-145 RB60U 60 nM BTZ 24 h
6→ DU-145 RB60 Untreated 24 h
7→ DU-145 RB60 60 nM BTZ 24 h
8→ X
9→ X

1→ DU-145 naïve Untreated
2→ DU-145 naïve 20 nM BTZ 24h
3→ DU-145 naïve 60 nM BTZ 24 h
4→ DU-145 RB60U Untreated
5→ X (cropped out)
6→ DU-145 RB60U 60 nM BTZ 24 h
7→ DU-145 RB60 Untreated 24 h
8→ DU-145 RB60 60 nM BTZ 24 h
9→ X

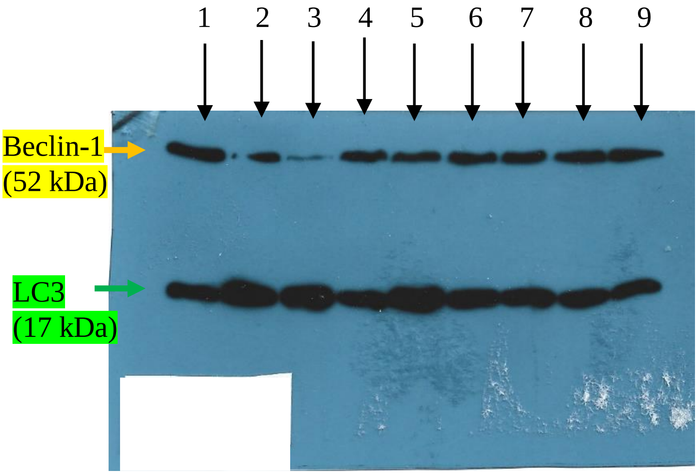

1→ DU-145 naïve Untreated
2→ DU-145 naïve 20 nM BTZ 24h
3→ DU-145 naïve 60 nM BTZ 24 h
4→ DU-145 RB60U Untreated
5→ DU-145 RB60U 60 nM BTZ 24 h
6→ DU-145 RB60 Untreated 24 h
7→ DU-145 RB60 60 nM BTZ 24 h
8→ X
9→ X

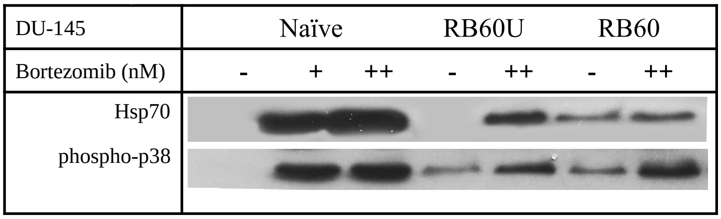

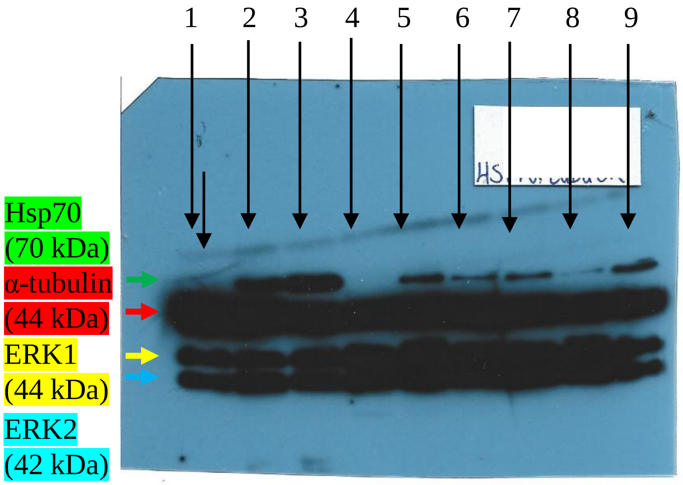

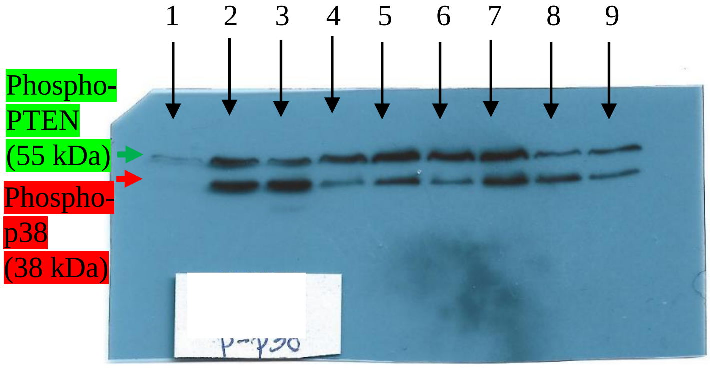

1→ DU-145 naïve Untreated
2→ DU-145 naïve 20 nM BTZ 24h
3→ DU-145 naïve 60 nM BTZ 24 h
4→ DU-145 RB60U Untreated
5→ DU-145 RB60U 60 nM BTZ 24 h
6→ DU-145 RB60 Untreated 24 h
7→ DU-145 RB60 60 nM BTZ 24 h
8→ X
9→ X

1→ DU-145 naïve Untreated
2→ DU-145 naïve 20 nM BTZ 24h
3→ DU-145 naïve 60 nM BTZ 24 h
4→ DU-145 RB60U Untreated
5→ DU-145 RB60U 60 nM BTZ 24 h
6→ DU-145 RB60 Untreated 24 h
7→ DU-145 RB60 60 nM BTZ 24 h
8→ X
9→ X
